# Complete mitogenome of three flyingfishes *Cheilopogon unicolor*, *Cheilopogon arcticeps* and *Cheilopogon atrisignis* (Teleostei: Exocoetidae)

**DOI:** 10.1080/23802359.2016.1144096

**Published:** 2016-03-28

**Authors:** Chang-En Chou, Feng-Yu Wang, Shui-Kai Chang, Hsueh-Wen Chang

**Affiliations:** a Department of Biological Sciences, National Sun Yat-sen University, Kaohsiung, Taiwan;; b Taiwan Ocean Research Institute, National Applied Research Laboratories, Kaohsiung, Taiwan;; c Institute of Marine Affairs, National Sun Yat-sen University, Kaohsiung, Taiwan

**Keywords:** *Cheilopogon*, long-PCR, mitochondrial genome, NGS

## Abstract

The complete mitogenomes of *Cheilopogon unicolor*, *C. arcticeps* and *C. atrisignis* were determined by the next-generation sequencing (NGS) method. The assembled mitogenome of *C. unicolor*, *C. arcticeps* and *C. atrisignis* consist of 16 529 bp, 16 530 bp and 16 530 bp, respectively. Three mitogenomes contain the typical gene complement including 13 protein-coding genes, 22 transfer RNAs, two ribosomal RNA genes and a non-coding D-loop. The length of D-loop is 870 bp (*C. unicolor* and *C. arcticeps*) and 869 bp (*C. atrisignis*), located between tRNA-Pro and tRNA-Phe. Phylogenetic analysis indicates that *Cheilopogon* is not monophyly. The mitogenomes of *C. unicolor*, *C. atrisignis* and *C. arcticeps* may provide useful information for phylogentic and population genetic analysis for flyingfishes.

The flyingfish is widespread in tropical and warm temperature waters. The most particular morphological character of flyingfish is its exceptionally large pectoral fins. The genus *Cheilopogon*, with most species and morphologically variable within flyingfishes, is not monophyletic (Lewallen et al. 2011) and no mitogenome data have been reported. We determined the complete mitogenomes of *C. unicolor*, *C. arcticeps* and *C. atrisignis* using specimens collected from waters off Pingtung and Yilan, Taiwan. Their genomic DNA was extracted from muscle using Wizard Genomic DNA purification Kit (Promega, Madison, WI). Long-PCR amplifications were performed by thermo-cycling using five pairs of primers and PCR amplicons were subjected to build up genomic library and pair-end sequencing by MiSeq (Illumina, San Diego, CA). Sequences were assembled using CLC Genomics Workbench version 7.0 (CLC Bio, Cambridge, MA). MitoFish (Iwasaki et al. 2013) were used to annotate the protein-coding and RNA genes.

Complete mitogenomes of *C. unicolor*, *C. arcticeps* and *C. atrisignis* consist of 16, 529 bp (GeneBank: KU360727), 16, 530 bp (GeneBank: KU360728) and 16, 530 bp (GeneBank: KU36072), respectively. These mitogenomes contain the typical gene complement including 13 protein-coding genes, 22 transfer RNAs, two ribosomal RNA genes and a non-coding D-loop. The overall base composition of *C. unicolor* is 29.1% for A, 27.3% for T, 27.2% for C, and 16.4% for G, of *C. arcticeps* 29.2% for A, 27.3% for T, 27.1% for C, and 16.3% for G, of *C. atrisignis* 28.8% for A, 27.2% for T, 27.3% for C, and 16.6% for G. In all three species, seven of 13 protein-coding genes terminate with incomplete stop codons of either T– (*ND2*, *COX2*, *ND3*, *ND4* and *Cytb*) or TA– (*ATP6* and *COX3*). Meanwhile, the longest protein-coding genes of these species was ND5 (1838 bp), whereas the shortest ATP8 (164 bp). The length of D-loop is 870 bp (*C. unicolor* and *C. arcticeps*) and 869 bp (*C. atrisignis*), located between tRNA-Pro and tRNA-Phe. The average number of nucleotide differences of three species is k: 1031.67, resulting in a nucleotide diversity Pi of 0.062.

**Figure 1.**
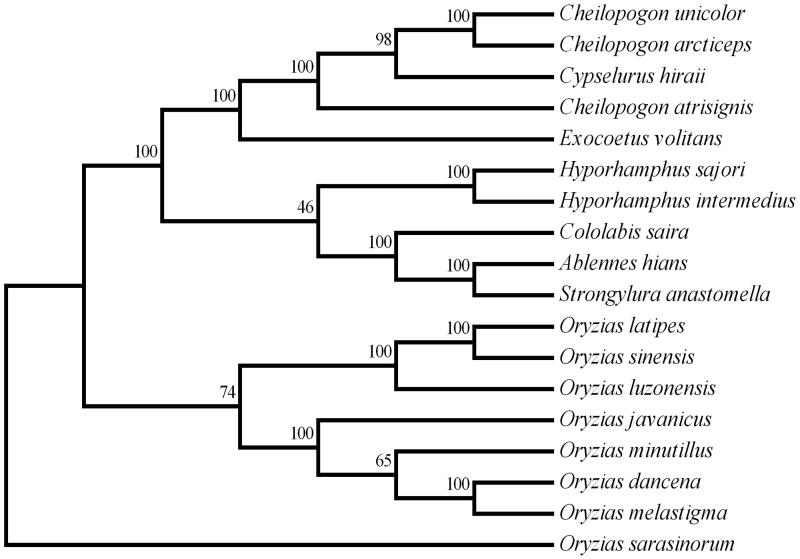
Molecular phylogeny tree of *Cheilopogon unicolor*, *C. arcticeps*, *C. atrisignis* and other 15 species in Beloniformes based on the complete mitogenome. The complete mitogenomes were downloaded from GenBank and the phylogenic tree was constructed by the Maximum parsimony method with 1000 bootstrap replicates. The gene’s accession numbers for tree construction are listed as follows: *Hyporhamphus sajori* (AB370892), *Hyporhamphus intermedius* (KP260625), *Strongylura anastomella* (KP864662), *Cololabis saira* (AP002932), *Ablennes hians* (AB373007), *Oryzias sinensis* (GU013788), *Oryzias sarasinorum* (AB370891), *Oryzias minutillus* (AB498068), *Oryzias melastigma* (JQ713914), *Oryzias luzonensis* (AB498064), *Oryzias javanicus* (AB498067), *Oryzias dancena* (AB498069), *Oryzias latipes* (AP004421), *Exocoetus volitans* (AP002933) and *Cypselurus hiraii* (AB182653).

To validate the phylogeny of *Cheilopogon*, we used MEGA 6 (Tamura et al. 2013) software to construct a Maximum parsimony tree (with 1000 bootstrap replicates) along with whole mitogenomes of 15 species of Beloniformes. Phylogenetic tree indicates that *Cheilopogon* is not monophyly and *Cypselurus hiraii* is closer to *C. unicolor* and *C. arcticeps* than *C. atrisignis* (Figure 1). The result is similar to that of the molecular phylogeny based on the Exocoetidae family that *Cheilopogon* belongs, and suggests that the systematics of *Cheilopogon* needs to be further clarified (Lewallen et al. 2011). We expect that the mitogenomes of *C. unicolor*, *C. atrisignis* and *C. arcticeps* may provide useful information for phylogentic and population genetic analysis for flyingfishes.
